# The various routes to functional regeneration in the central nervous system

**DOI:** 10.1038/s42003-020-0773-z

**Published:** 2020-01-29

**Authors:** Karen Echeverri

**Affiliations:** Eugene Bell Center for Regenerative Biology and Tissue Engineering, Marine Biological Laboratory, Woods Hole, MA USA

**Keywords:** Experimental organisms, Regeneration, Spinal cord injury

## Abstract

The axolotl is a type of Mexican salamander with astonishing regenerative capacity^1^. In our recent paper, we identified a signaling heterodimer that is formed directly after injury in the glial cells adjacent to the injury in axolotls. The c-Fos and JunB genes forming this heterodimer are not unique to animals with high regenerative capacity but they are present in humans too. In this paper I propose perspectives on molecular control of regeneration and future directions that need to be taken to advance our understanding of regeneration at a molecular level.

## Abstract (web summary)

Karen Echeverri and her colleagues showed in their recent Communications Biology study that an unconventional Fos/Jun heterodimer regulates axon regeneration in axolotl. In this article, she emphasises the diverse mechanisms of regeneration in other species and discusses future work needed to understand how the nervous system can be regenerated.

## Diversity among species in mechanisms of regeneration

The ability to functionally regenerate is a phenomenon that has fascinated mankind for centuries. Aristotle (384–322 BC) made one of the first written observations of lizards regenerating their tails, yet centuries later the molecular blueprints for this fascinating skill set remain elusive^2^. Many animals and plants display various degrees of regenerative capacity and interestingly, there are many roads to the same endpoint even amongst highly related species^3^. Salamanders, for example, can functionally regenerate their limbs, which includes muscle fibers. The terrestrial newt does this by dedifferentiating its mature muscle to form mononucleate cells that act as progenitors for the new muscle, whereas the highly related aquatic Mexican salamander, the axolotl, utilizes its Pax7 positive satellite cells to regenerate its muscle fibers^4^. Why such closely related species use such different mechanisms to regenerate the same cell type is unknown, but it shows the importance of studying a wide range of research organisms to understand and eventually define the principles of regeneration.

Humans have very restricted regenerative capability but we can repair lesions in our muscle using endogenous satellite cells, similar to those found in highly regenerative species like axolotls, *Parhyale* and zebrafish. We can also repair lesions in our peripheral nervous systems; if our muscle is damaged, the nerves innervating that tissue are often also damaged and we can repair these types of injuries up to a certain size. However despite the ability to regenerate peripheral nerves, the scenario in the central nervous system is very different. After injury to the spinal cord in humans, surrounding cells, like glial cells, fibroblasts and immune cells, respond to the initial injury signal cues and migrate to the injury site. The injury activates a process termed reactive gliosis and those cells which migrate to the injury site start to express proteins that they normally don’t express and come together to form a glial scar. This glial scar has both positive and negative aspects—it is thought to prevent further injury but has also been shown to express proteins that inhibit axon regeneration^5–8^.

## Similarities and differences in spinal cord regeneration

The spinal cord presents a similar diversity by which a specific animal will reach a functional end point. Lamprey, the most basal vertebrate that regenerates neurons, can regenerate after complete spinal cord transection and research from several laboratories has shown that its central nervous system consists of “good regenerators and bad regenerators”. However despite not all neurons having the capacity to regenerate, Lamprey can regain functional locomotive activity^9–14^. Work in zebrafish has shown a similar phenomenon, although exactly what percentage is regenerating is harder to define due to a larger number of neurons^15,16^. Axolotls are thought to, in fact, regrow all of their neurons after complete transection of their spinal cord^17^. This could be related to differences in how the severed neural tube regenerates. For example, in zebrafish, the glial fibrillar acidic protein (GFAP)-positive glial cells lining the central canal delaminate and stretch out to bridge the injury site^18,19^. Axolotls activate glial cell division and migration to fill in the missing portion of the neural tube, such that it is morphologically impossible to distinguish old from new tissue (Table 1)^20^. Lamprey and *Xenopus* appear to have quite different glial cells to other animals in that both animals seemingly lack a true GFAP in their genome. This may be why no true glial scar is formed in these animals after injury and hence a more permissive injury environment is presented to the severed neurons^21–23^. In Lamprey it would then suggest that intrinsic differences within neurons determine their regeneration capacity. In *Xenopus* the situation is more complicated. In the larval stages they can regenerate the spinal cord; glial cells adjacent to the injury upregulate the neural progenitor marker sox2, similar to axolotls and these cells migrate and divide to repair the injury. However after metamorphosis regenerative ability is lost^24–26^. This has been linked to the maturation of the immune system, which is known to play a role in reactive gliosis in humans (22–24).

**Table 1:** A summary of the current state of data regarding glial cells and composition of the AP-1 factor activated in them after spinal cord injury.

Species	Functional CNS Regeneration	GFAP + Glial cells	AP-1 components activated after injury	References
Humans	No	Yes	c-Fos, c-Jun	^5,7,8,27^
Axolotls	Yes	Yes	c-Fos, JunB	^1^
Zebrafish	Yes	Yes	Fos, Jun	^19^
Xenopus	Larvae only	No	Fos, Jun	^28,29^
Lamprey	Yes	No	Fos, Jun	^23^

In many species the exact Fos or Jun family members are not specifically identified in transcriptional profiling data

## Axolotl glial cell activation

In our recent paper we have begun to examine at a molecular level how axolotl glial cells are activated to divide and migrate in response to injury. We have identified a heterodimer consisting of c-Fos and JunB which is transiently activated and which functionally regulates the GFAP promoter preventing up-regulating GFAP expression. The c-Jun gene is present in axolotls and is repressed in response to injury by activation of miR-200a. Overexpression of c-Jun in the axolotl glial cells leads to upregulation of GFAP and other genes involved in reactive gliosis and ultimately blocks axon regeneration. In mammals, work from many groups have revealed that after spinal cord injury, glial cells show a prolonged upregulation of the canonical AP-1 transcription factor composed of the heterodimer c-Fos and c-Jun which instead activates the GFAP promoter^27^. This work illustrates that small changes in heterodimer formation can lead to substantially different outcomes probably due to very different signaling pathways activated downstream. Our research also brings up many interesting questions and potential avenues to follow. Recently we have tested whether changing the composition of the heterodimer can affect the mammalian GFAP promoter and indeed, preliminary in vitro experiments suggest that the non-canonical c-Fos:JunB heterodimer formed in axolotl after injury fail to induce high activation of the GFAP promoter. We have also begun to examine other species with high regenerative capacity to ask upregulation of the non-canonical AP-1 ^c-fos:JunB^ occurs. Initial data-mining of publicly available RNA sequencing data shows up-regulation of Fos and Jun family members in many regenerative species. However due to lack of high-level annotation of many genomes it is often unclear exactly which family member is activated in these data sets. Nevertheless, it is important to note that although Lamprey and *Xenopus* do not appear to have a GFAP gene, they activate Fos and Jun family members after injury^23,28,29^. The functional significance of this activation remains to be elucidated. It’s likely that injury-induced genes modulate several pathways to direct the response to injury in the spinal cord.

One of the future challenges is to determine, in axolotl, which are the important signaling pathways downstream of the GFAP promoter as well as identifying other potential pathways affected by the heterodimer. In our recent paper we carried out RNA sequencing on the axolotl spinal cord tissue 4 days post injury providing a wealth of data to be mined. As expected, markers of neuronal differentiation and synaptic signaling are downregulated after injury; however when miR-200a is inhibited we see mis-regulation of classical markers of neuronal differentiation and axon guidance. This could suggest that miR-200a may not just regulate c-Jun but also other target genes involved in regulating the differentiation state of cells after injury, and potentially some cells may revert to a more neural stem cell-like state in the axolotl after injury.

## The role of the immune system

In addition, genes involved in the immune system are highly upregulated in regeneration. There has been renewed interest in the role of the immune system in pro-regenerative versus non-regenerative species in recent years. Earlier work in *Xenopus* limb regeneration suggested that the maturation of the immune system potentially was a large causative factor in the inability of the frog to regenerate after metamorphosis. However biology is rarely binary and work from many labs has shown that there is also a need for the immune system in regeneration. Ultimately it may all be about timing and duration of the immune stimulation. Recent work has very elegantly illustrated this aspect in two closely related species that have stark regenerative (Zebrafish) and non-regenerative (Medaka) responses to injury to the heart. In zebrafish recruitment of macrophages is necessary for activation of the regenerative program but it is not an absence of macrophages in Medaka that is responsible for its lack of regenerative ability, rather, an interesting difference in the timing and duration of immune cells recruited to the injury site^30^.

What pathways actually recruit immune cells to the injury site and control their dynamics is a big question that needs to be addressed. Data from many research organisms, especially zebrafish (because of the availability of transgenic lines for different immune cells) has provided interesting insights into the types of immune cells recruited to different types of injuries and their dynamics^31^. The next level is understanding their molecular control in detail. To date many signals like TGF-beta, Toll receptors and interleukins are know to be involved in recruiting immune cells. The AP-1 transcription factor has also been implicated. Interestingly JunB interacts with IL-1ß and with ATF3, genes with known roles in the immune system. This might suggest that in animals that lack a GFAP gene, Fos and Jun play roles in modulating the immune response in the spinal cord after injury.

## Future outlook

The far-reaching goal of studying nervous system regeneration in species that can do it naturally is that one day this knowledge can inform strategies for therapies for patients with spinal cord injury and neurodegenerative diseases. It is clear from studying a small snapshot of organisms with functional regenerative capacity that there is no single pathway to regeneration. One question that scientists are often faced with is “how similar” are axolotl glial cells to human cells. To date we know from transcriptional profiling studies that glial cells from axolotl or zebrafish express many of the same genes as human cells. In the future it will be interesting to determine if the regulatory elements of these genes have evolved differently and whether they determine the initial response to injury in order to direct cells towards a certain pathway. Research from many groups using different organisms clearly shows that nature has evolved many different routes to regenerate functional spinal cords; however more in-depth knowledge is necessary to build blueprints for any one organism’s individual regenerative strategy.
